# Surgical Treatment in Silicone Oil-Associated Glaucoma

**DOI:** 10.3390/diagnostics12041005

**Published:** 2022-04-16

**Authors:** Catalin Cornacel, Otilia-Maria Dumitrescu, Alexandra Catalina Zaharia, Ruxandra Angela Pirvulescu, Mihnea Munteanu, Calin Petru Tataru, Sinziana Istrate

**Affiliations:** 1 Ophthalmology Department, “Dr. Carol Davila” Central Military Emergency University Hospital, 010825 Bucharest, Romania; catalincornacel@gmail.com (C.C.); alexandra.zahariac@gmail.com (A.C.Z.); 2 Department of Ophthalmology, “Victor Babes” University of Medicine and Pharmacy, 300041 Timisoara, Romania; mihneam1@gmail.com; 3 Department of Ophthalmology, “Carol Davila” University of Medicine and Pharmacy, 050474 Bucharest, Romania; ruxandra.pirvulescu@umfcd.ro (R.A.P.); calinpetrutataru@yahoo.com (C.P.T.); sanzinici@yahoo.com (S.I.)

**Keywords:** silicone oil, glaucoma, surgical treatment, trabeculectomy, Ahmed valve, cyclophotocoagulation, Ex-PRESS minishunt

## Abstract

Glaucoma is a vision threatening, not uncommon complication of eyes that have undergone pars plana vitrectomy with silicone oil endotamponade. Although most patients respond well to medical antiglaucoma therapy, there are refractory cases where surgery is required to control the intraocular pressure. This review, following a comprehensive literature search in the Medline database, aims to present the most important surgical techniques currently in use for glaucoma associated with silicone oil endotamponade and their indication depending on the mechanism of glaucoma. In cases of pupillary block, the presence of a patent iridotomy or iridectomy must be ensured, either by laser or surgically. When silicone oil is in excess and whenever the retinal status permits it, partial or complete removal of the silicone oil should be performed. Trabeculectomy has shown higher failure rates and more complications in these cases compared to other indications, so alternate methods are warranted. For very high intraocular pressures, glaucoma drainage devices and transscleral cyclophotocoagulation are the most used options, with good efficacy and safety profiles, although rarely they may have serious complications. The Ex-PRESS mini shunt has shown excellent results and lower rates of complications. For less important IOP elevations, minimally invasive glaucoma surgery and selective laser trabeculoplasty may be used, either alone or in conjunction with other methods.

## 1. Introduction

Silicone oil (SO) is an endotamponade agent widely used in conjunction with pars plana vitrectomy (PPV) and is the agent of choice in complex cases, such as retinal detachments in the setting of proliferative vitreo-retinopathy, proliferative diabetic retinopathy, trauma, recurrent retinal detachments, viral retinitis, as well as in cases of giant retinal tears and macular hole repair [1,2,3,4,5].

Owing to surface tension and volume displacement, SO acts by pushing the retina peripherally and applying it to the eye wall, while also sealing retinal breaks and preventing retinal redetachment [2]. SO provides extended, even permanent tamponade [3]. Moreover, it serves as a barrier against the spread of inflammatory cytokines and of proliferating cells. SO also has a hemostatic function and a protective effect against the development of rubeosis iridis [2]. Having a stable volume, it is more versatile than gas tamponade, as it does not require strict positioning of the patient in the postoperative period and does not contraindicate air travel [2]. Its optical properties also allow a clear view of the fundus, facilitating the follow-up of the patients [3]. The specific gravity of conventional SO of 0.97 g/cm^3^ makes it lighter than water, thus, when present in the anterior chamber (AC), it assumes a superior position, while the aqueous humor is displaced inferiorly [2]. In 2003, “heavier than water” SO was introduced for the treatment of inferior retinal breaks, owing to the fact that it assumes an inferior position [2,6]. 

In spite of its obvious advantages, which make it so widely used in vitreo-retinal surgeries, SO endotamponade has been associated with multiple complications, some of the most important being cataract formation, increased intraocular pressure (IOP), keratopathy (chronic or recurrent corneal edema, band keratopathy), migration of SO into the AC, emulsification of SO present in the AC, migration of SO into the subretinal space through retinal breaks, inflammation with membrane formation, iritis [2,3,7].

Increased IOP is the second most common complication of PPV with SO tamponade after cataract formation, with a prevalence of 2.2–56% [8,9,10]. Making the diagnosis of glaucoma in these cases is not always easy and glaucoma may silently lead to vision loss. IOP may be difficult to measure, because of an altered corneal status, as is also the case in anterior segment pathology or after corneal surgery [11,12,13,14]. Moreover, decreased visual acuity with associated impaired fixation may preclude IOP measurement, similarly to ocular media opacities and advanced macular disease [15,16,17]. What is more, a correct evaluation of the optic nerve is not always possible due to poor fixation and difficult visualization of the retina. In the majority of cases, the postoperative increase in IOP can be managed medically [3,4,10,18]. However, when antiglaucoma medication is inefficient, surgical management is warranted and intervention should be prompt and tailored to the specific mechanism of IOP elevation.

## 2. Materials and Methods

We conducted an extensive literature search in the Medline electronic database, using the PubMed interface. The search process comprised the following word combinations: either “surgery” or “surgical treatment” AND “silicone oil” AND either “ocular hypertension” or “glaucoma”. Inclusion criteria consisted in articles written in English, regarding human pathology and subjects, that had appeared before March 2022. We evaluated the title and abstract and retained studies that described and/or compared surgical procedures used in the management of silicone oil-associated ocular hypertension or glaucoma, as well as studies that explored the prevalence and pathophysiology of glaucoma related to silicone oil tamponade. Additional references were obtained from the reference lists of the already included studies. We excluded studies that were not written in English, that were duplicates of the previously retained ones, as well as editorials, letters to editors, comments, conference presentations, and articles that were not relevant to the topic. We retained 62 articles, dating from 1986 to 2022.

## 3. Literature Review

### 3.1. Silicone Oil-Associated Ocular Hypertension and Glaucoma

In a study that included 2997 patients with newly diagnosed glaucoma, conducted in a tertiary glaucoma center in India in 2005, vitreoretinal surgery was found to be the most frequent cause of secondary glaucoma, especially in the 21–60 age group. Of all post-vitrectomy patients that presented with glaucoma, 73% had silicone oil, with 60% having the endotamponade for more than 3 months [19]. In this context, two distinct entities have been identified: early onset ocular hypertension (OHT) and late-onset glaucoma [2].

Early onset OHT is not a single entity, but rather encompasses multiple postoperative states than can lead to increased IOP. The underlying mechanisms can be related to angle closure or can act in the presence of an open angle. Angle closure usually occurs due to pupillary block resulting from the migration of SO from the posterior pole to the AC or from the presence of extensive posterior synechia. Other mechanisms of angle closure include peripheral anterior synechia and the contraction of the fibrovascular membranes present in the angle in neovascular glaucoma. Early onset increased IOP associated with an open angle can occur in the setting of pre-existing open angle glaucoma (OAG), important inflammation, mechanical blockage of the trabecular meshwork by the SO present in the AC and postoperative steroid treatment [20]. Heavier than water SO is more prone to emulsification compared to standard SO and emulsification occurs earlier. Emulsified droplets are responsible for mechanically blocking the trabecular meshwork, as well as for an inflammatory reaction, both of which result in increased IOP [2].

Late-onset glaucoma is an important source of ocular morbidity and may lead to blindness. A study by Lyssek-Boroń et al. found that the risk of consistently raised IOP was 4.7 times higher when SO was used as a tamponade agent than when balanced saline solution and sulfur hexafluoride were used instead [18]. Pre-operative risk factors include aphakia, diabetes mellitus, pre-operative increased IOP, uveitis, trauma and previous vitreo-retinal surgery [4,8,10,21,22]. Post-operative risk factors include the presence of SO in the AC, SO emulsification, rubeosis iridis, hyphema, peripheral anterior synechia, and chronic steroid therapy [4,10,20,22]. The mechanisms involved consist in angle closure through PAS or neovascular membranes, inflammation and erythroclastic glaucoma [3,9,23,24]. Inflammation appears to be an important mechanism for glaucoma related to SO endotamponade. Liu et al. found that proinflammatory mediators, such as IL-17 and its effector molecules, IL-6 and TNF-alpha, have significantly higher levels in the aqueous humor of patients who have secondary glaucoma, than in patients with SO without glaucoma. TNF-alpha promotes apoptosis and is involved in the trabecular meshwork dysfunction [25]. However, in the late postoperative period, glaucoma is most often the result of the emulsification of SO present in the anterior chamber, resulting in silicomacrophagocytic open-angle glaucoma [5]. SO emulsification occurs in 0.7–56% of cases and is usually apparent on gonioscopy [3,4,25,26,27]. The risk of SO emulsification is increased in aphakic eyes and after trauma (due to zonular dehiscence leading to SO migration into the AC) and is promoted by the use of lower viscosity SO, by ocular movements in SO-underfilled eyes (which results in mechanical emulsification) and by contact with various molecules, such as perfluorocarbon liquids and other biological emulsifiers [3,6,18,26]. The rate of emulsification appears to be proportional to the duration of the endotamponade [18,26]. SO-induced glaucoma is often rapidly and silently evolving, which may be additionally explained by the direct neurotoxic effect that SO exerts on the optic nerve fibers [6] and by the blockage of nutritional and signaling factors that usually circulate in the vitreous cavity [25].

### 3.2. Surgical Management of Pupillary Block and of Angle Closure

Pupillary block is the most common mechanism of angle-closure glaucoma associated with SO tamponade. SO that migrates anteriorly displaces the aqueous humor, which accumulates inferiorly in the posterior chamber. On the one hand, this results in pushing the iris anteriorly with a subsequent irido-trabecular contact and, on the other hand, it allows for more SO to enter the AC until SO completely fills the AC and the pupil and accumulates superiorly in the posterior chamber [2]. The migration of one large SO bubble can also produce iridolenticular block, especially with the use of heavy SO in patients adopting prolonged supine positioning [5,28]. SO migration from the vitreous cavity to the anterior segment occurs more frequently in aphakic eyes, but it may be seen in pseudophakic and phakic eyes as well, if there are areas of zonular disinsertion which allow the passage of SO [3,29].

PPV with SO tamponade in aphakic eyes should be associated with a prophylactic inferior surgical iridectomy at 6 o’clock, for the prevention of pupillary block [2,30]. Some authors expand this indication to pseudophakic eyes [30,31]. In cases where “heavier than water” SO is used, the iridectomy should be placed superiorly, at 12 o’clock [2]. However, closure of the prophylactic iridectomy is not uncommon, occurring in 11 to 32% of patients [2]. Inflammation in the presence of SO can lead to the formation of retroiridal inflammatory membranes or to fibrin clots in the anterior chamber that occlude the iridectomy [3,30]. In the presence of acute angle closure, IOP must be immediately lowered, initially medically, with aqueous suppressants and osmotic agents [2,32]. As soon as possible, the iridectomy must be reopened, or, if it does not already exist, it should be created, just inferior to the SO meniscus [33]. In both cases, the Nd:YAG laser or a combined Argon-Nd:YAG laser can be used to reestablish aqueous humor flow between the posterior and anterior chambers [9,30]. Multiple treatment sessions may be necessary over time, as, although initial patency can be obtained in the majority of cases, iridectomy/iridotomy failure is common [29,31]. Additional adjunctive measures have also been proposed, such as subconjunctival or sub-Tenonian administration of corticosteroids or intracameral tPA, with the aim of reducing inflammation and dissolving the fibrin clot [30]. When laser is ineffective, surgical opening of the iridectomy should be attempted [2]. A transfixion technique for the treatment of silicone oil pupillary block glaucoma refractory to Nd-YAG iridotomy using a 30G needle passed through the corneoscleral limbus was described. The procedure was successful in restoring the flux of aqueous through the iridectomy and in pushing the SO back into the vitreous [34]. Pupillary dilation may also be useful, as it reduces the risk of recurrent attacks [28]. If the risk of retinal redetachment is low, SO removal can be considered [28].

In cases of angle closure related to extensive PAS formation, breaking the PAS is the appropriate solution when IOP is uncontrollable medically. Narang et al. described a technique of single-pass four-throw pupilloplasty for breaking the PAS, that was successful and was not associated with significant complications. SO was removed prior to pupilloplasty, as part of the same surgery. An additional advantage of the procedure is that it allows adequate pupillary dilatation for retinal examinations [35].

### 3.3. Silicone Oil Removal

SO endotamponade is usually maintained for 3 to 6 months postoperatively, but in cases where there is increased risk of retinal redetachment, it can be left in place as long as the surgeon deems necessary, even indefinitely [6]. Honavar and colleagues found that 70% of the glaucoma cases occurring after PPV with SO endotamponade can be directly attributable to SO [4]. Consequently, SO removal appears as a rational option if the risk of retinal redetachment does not exceed the benefits of IOP lowering through SO removal. Usually, this implies having a completely attached retina, all retinal tears closed and no areas of retinal traction [36]. SO can be removed entirely or partially. If SO overfill is present, this should be recognized and addressed as soon as possible by the surgeon, by removing the SO excess [1].

In a study that compared SO extraction alone, glaucoma surgery alone and the association of the two, Budenz et al. found that SO extraction alone resulted in a 62% success rate at 24 months, compared to a 50% success rate in the case of concurrent SO removal and glaucoma surgery. The authors noted that, in the SO extraction alone group, failure was due mostly to postoperative uncontrolled high IOP (92% of cases), while in the group that associated two procedures, it was mostly the result of hypotony (75% of cases) [1]. Other reported IOP control rates after SO removal are Nguyen et al.—57.14% [37] and Honavar and colleagues—45.5%, but in the latter, all patients still required IOP lowering medication [4]. Jonas et al. reported a significant reduction in IOP after SO removal, which appeared proportional to the IOP value. In the same study, after SO removal, patients whose IOP did not return to values within the normal range, were managed medically [5]. Complications of SO removal include hypotony, retinal detachments, and rubeosis iridis [5].

Selective removal of the SO present in the AC is a good alternative, as it allows continuation of the endotamponade and thus reduces the risk of retinal redetachment. In their technique, Wahab et al. removed the SO from the AC by gradually filling the AC with air by the use of a vitreo-retinal surgery air pump, while at the same time allowing the SO to either fall posteriorly to the posterior segment or to exit the eye through a paracentesis kept open by pressing on its posterior lip [38]. A risk arising from partial SO removal in the supine position is increased migration of SO from the vitreous cavity into the AC, following its tendency to float [39]. On the one hand, this makes SO removal from the AC inefficient and, on the other hand, increases the risk of retinal redetachment, as SO leaves the vitreous cavity. Using a similar technique, Baskaran et al. showed that concomitant and continuous air infusion into the AC prevents SO from rising anteriorly. At the conclusion of surgery, sideports were closed with 10-0 nylon sutures in order to prevent loss of air and facedown positioning was prescribed for one week postoperatively. Possible complications include endothelial damage and Descemet membrane detachment [39].

However, SO removal does not guarantee IOP control. Moisseiev and colleagues reported achieving control of the IOP by SO extraction alone in only one of their 11 patients [40]. This is probably due to the fact that prolonged contact between SO and the trabecular meshwork induces fibrosis and trabecular meshwork collapse [4]. If changes at the level of the trabecular meshwork have already occurred and result in IOP elevation, other methods of IOP lowering must be considered.

### 3.4. Trabeculectomy

Trabeculectomy is the surgical procedure of choice in cases with visual potential and refractory glaucoma [4]. However, trabeculectomy in the setting of SO endotamponade is less likely to achieve control of the IOP and more prone to failure, with hypotony being an important cause of postoperative failure [41]. This is mostly due to less available naïve conjunctiva and to the presence of emulsified SO, which induces inflammation and fibrosis at the level of the trabecular meshwork and in the subconjunctival space [37,42]. Trabeculectomy is usually performed in conjunction with mitomycin C (MMC) application in order to reduce fibrosis [42]. The surgery is technically difficult, because of conjunctival scarring resulting from the previous PPV [37]. Moreover, the conventional superior placement of the bleb makes the ostium prone to blockage by the SO present in the AC [4]. As a result, if SO is found in the AC or in the angle during gonioscopy, SO removal prior to trabeculectomy may be indicated [42]. The alternative of an inferiorly placed trabeculectomy is discouraged because of the increased risk of infectious complications [4]. Complications associated with trabeculectomy include hyphema, hypotony, serous choroidal detachment, recurrent retinal detachment, failure of filtration with persistent ocular hypertension and, more rarely, blebitis and endophtalmitis [43,44]. Bleb needling may be required to maintain filtration [42,43].

In one study, Errico et al. compared trabeculectomy with MMC with the Ex-PRESS glaucoma filtration device in eyes in which SO had been extracted. They found that, at two years, in the trabeculectomy group, the complete success rate (controlled IOP without antiglaucoma medication) was 40% and the qualified success rate (controlled IOP with glaucoma medication) was 60%, compared to success rates of 73% and 81.8%, respectively, in the Ex-PRESS group [43]. El-Saied and colleagues also reported the inferiority of trabeculectomy (50% success rate) to Ahmed valve implantation (80% success rate) and Ex-Press minishunt (100% success rate), even though statistical significance was not reached [44]. Honavar et al. achieved good control of IOP in 3 out of 5 patients treated with trabeculectomy after SO removal [4]. The study by Singh et al. showed a qualified success rate (IOP lower than 21 mm Hg with antiglaucoma medication, needling or both) of 21.1% at one year and an absolute success rate (IOP lower than 21 mm Hg in the absence of medication or needling) of 15.8% at one year. The authors also found that the success rate at three months postoperatively is similar to that at 12 months and that failure occurred mostly in the first 6 months post-trabeculectomy. Thus, they inferred that the early behavior is an important indicator of the long-term success. Owing to the poor success rates, the study concluded that the search for alternatives to trabeculectomy is necessary in the case of glaucoma associated with SO tamponade [42].

### 3.5. Glaucoma Drainage Devices

Glaucoma drainage devices have gained increased popularity in the treatment of medically unresponsive glaucoma, and about one in 1000 persons per year requires the implantation of a glaucoma drainage device after vitreo-retinal surgery [45]. The Ahmed glaucoma valve implant is the most frequent choice, but non-valved devices, such as the Molteno and the Baerveldt have also been successfully used. Albahlal and colleagues reported a 94.1% success rate and a significant reduction in the number of antiglaucoma medications at one year, using both unvalved (Baerveldt glaucoma implant and Aurolab Aqueous Drainage Implant) and the Ahmed glaucoma valve implant [23]. In their series, most of the patients had already had the SO removed, which may explain the high success rate. In another study, Nguyen et al. achieved a successful reduction in IOP in 3 out of 5 patients using a Molteno implant [37].

The Ahmed glaucoma valve (AGV) has been proved capable of providing IOP control in most cases of glaucoma associated with SO [46]. Success rates reported in different studies vary between 62% and 80%, at follow-up intervals usually between 12 and 24 months [41,44,46,47]. Success rates and the reduction in the mean IOP obtained by different studies are shown in Table 1. AGV implantation also resulted in a significant reduction in the number of antiglaucoma medications [44,46,47].

The presence of SO increases the risk of failure of AGV, either through direct blockage of the drainage pathway or through the migration of SO into the subconjunctival space, where it promotes chronic inflammation and fibrosis [41,46]. In the presence of SO, prolonged use of steroids is usually required [46,47]. Complications are frequent and can range from mild and easily manageable, such as a postoperative hypertensive phase, hyphema or low-grade inflammation, to more serious ones, such as hypotony (in up to 50% of patients [44]) and choroidal effusion, tube-cornea touch, eye motility disorders with inferotemporal placement of the valve (the last two complications requiring repositioning). Vision threatening complications have also been reported and include tube or plate exposure with subsequent endophthalmitis (more frequent in younger patients), bullous keratopathy, retinal redetachment, and even phthisis bulbi [41,44,46,47].

Superior placement of the AGV makes it susceptible to blockage by SO [46,48]. Inferior placement, less prone to SO obstruction, has been performed with good results [46,47]. The inferonasal quadrant appears to be safer with regard to complications [46,47]. Intraoperatively, viscoelastic may be used to prevent migration of SO anteriorly from the vitreous cavity and into the tube, and may even be left in place at the conclusion of surgery with no postoperative hypertonia [46,47]. In order to avoid exposure of the tube or plate, careful closing of the conjunctiva is warranted and, if the conjunctival tissue is insufficient, the use of patch grafts (corneal, scleral, or from other regions, such as the fascia lata), or of local scleral flaps may be employed [23,41,49]. Moreover, an oblique trajectory of the tube within the scleral tunnel also reduces the risk of exposure [23,41]. In an attempt to minimize the rate of complications, Davo-Cabrera et al. describe a technique that consists in positioning the tube in the posterior, rather than in the anterior chamber, which eliminates the risk of corneal touch and reduces SO migration through the tube [49].

In light of the poor results of trabeculectomy in SO-associated glaucoma, glaucoma drainage devices appear to be good alternatives, especially in eyes with good visual potential and high IOPs where an important and sustained decrease in IOP is warranted.

### 3.6. Transscleral Cyclophotocoagulation

Cyclodestructive procedures, especially transscleral, are of great interest in the setting of glaucoma associated with SO endotamponade. First, they do not depend on conjunctival integrity, as does conventional surgery. Second, they are not influenced by the presence or duration of SO endotamponade and so are a good alternative in cases where the risk of retinal redetachment is elevated and SO endotamponade must be maintained [50]. Third, owing to the non-invasive nature of transscleral approaches, there is minimal risk of infection and retreatment is possible. Currently, diode-laser transscleral cyclophotocoagulation (TSCPC) is the most used method and can be further subdivided into continuous wave (CW-TSCPC) and micropulse (MP-CPC); the latter having the advantage of less collateral damage to adjacent tissues [51]. Another technique, slow coagulation CW-TSCPC, which consists in delivering a fixed, lower level of energy over a longer period of time, has also been described [52]. The last two methods have less potency, but have better safety profiles [51,52]. All methods have proved to be efficient in significantly reducing IOP in eyes post-PPV with SO. Success rates were variable: Sivagnanavel et al. [53]—44%−, Albahlal et al. [23]—53.8%−, Khodeiry et al. [52]—72.2%−, Han et al. [22]—81.8% with antiglaucoma medication and 54.5% without additional antiglaucoma medication. Retreatment is often necessary to control IOP, with up to four sittings at intervals of six to eight weeks [50,54]. TSCPC also significantly reduces the number of antiglaucoma topical medications required for IOP control [22,53,54] and the use of oral acetazolamide [50,53]. The evolution of IOP and of the antiglaucoma medication in different studies is shown in Table 2.

TSCPC is often reserved for eyes with poor visual potential, for fear of complications and because it was not shown to protect against vision loss [53]. Possible complications are rare, but potentially serious, and include conjunctival burns, neurotrophic keratitis, scleritis, AC inflammation, hyphema, postoperative IOP spike, intravitreal hemorrhage, cystoid macular edema, retinal detachment and choroidal effusion [51,52,53,54]. The most feared complications are hypotony, which may be important and even lead to phthisis bulbi and, extremely rarely, sympathetic ophthalmia. Reported hypotony rates vary between 5.6% and 13.2% [50,52,53], while Han et al. and Kumar et al. reported no cases of hypotony in their series [22,54]. Worsening of visual acuity and even loss of light perception after the treatment are not rare (56% in a study by Sigvananavel et al. [53]), but they are frequently the result of the evolution of the glaucomatous optic neuropathy or of the underlying retinal condition [22]. Complications are more frequent with CW-TSCPC than with the other methods.

TSCPC is a good solution for eyes with SO endotamponade and glaucoma, as it is minimally invasive, effective, repeatable, and relatively safe.

### 3.7. Other Methods

#### 3.7.1. Ex-PRESS^®^ Mini Shunt

The Ex-PRESS^®^ mini shunt (Alcon Inc., Fort Worth, TX, USA), is a filtration device that has been successfully used in the treatment of open angle glaucoma secondary to SO emulsification. The tube is usually placed in the AC, but a pars plana approach has also been described [55]. Errico et al., who compared it to trabeculectomy, found complete success rate (without antiglaucoma medication) at 2 years of 73%, compared to 40% in the case of trabeculectomy [43]. The mean IOP decreased from 33 ± 6.4 mm Hg preoperatively to 17.8 ± 6.2 mm Hg at 2 years postoperatively [43]. El-Saied et al. reported a 100% success rate and no associated complications for the Ex-PRESS, with better results than trabeculectomy, deep sclerectomy, and AGV [44]. Most studies report no associated complications and postoperative needling is more rarely needed compared to trabeculectomy [43,44,55].

#### 3.7.2. Gonioscopy-Assisted Transluminal Trabeculotomy (GATT)

GATT is a minimally invasive glaucoma surgery that can be a good primary or adjunctive option in SO-associated glaucoma, before or after SO removal, with open angles without broad PAS [56]. It significantly reduced the IOP from 31 ± 4.1 mm Hg to 15.6 ± 4.6 mm Hg in a study by Aktas et al. [57] and from 32.7 ± 5.1 mm Hg to 13.6 ± 1.8 mm Hg in a study by Quan et al. [58], with a success rate of 93.3%, but with most patients requiring additional antiglaucoma medication [57]. GATT also significantly reduced the required number of antiglaucoma medications [58]. This method carries a good safety profile and has the advantage of sparing the conjunctiva thus permitting future bleb-forming procedures [58].

#### 3.7.3. Selective Laser Trabeculoplasty (SLT)

SLT can be used in open angle glaucoma associated with SO and IOP up to about 25 mm Hg as it was shown to reduce IOP by approximately 20% at one year, with an overall success at 12 months of 59.5% [59]. In the few studies that evaluated its use in this setting, SLT significantly reduced both the IOP at all follow-up visits up to 6 months [60] and, also, the number of antiglaucoma medications required to control the IOP [59].

#### 3.7.4. Supraciliary Shunts

Supraciliary shunts, such as the Cypass, which is no longer in use, and the gold micro shunts are attractive options that have proved effective in single case reports in SO-associated glaucoma [61,62]. However, their use in this particular setting has not been well explored. Moreover, their use has been associated with endothelial cell loss and with fibrosis and encapsulation [62] and the consequences of potential migration of SO in the suprachoroidal space are unknown [61]. More studies are required to establish their efficacy and safety profile.

## 4. Conclusions

Managing medically resistant IOP elevations in patients who have undergone PPV with SO endotamponade is a challenge for ophthalmologists. Depending on the mechanism of IOP rise, multiple choices are available. If pupillary block is present, a patent laser or surgical iridotomy or iridectomy should promptly be created. If there is SO overfill or if the risk of retinal redetachment is low, partial or complete removal of SO may be performed. SO removal is especially useful in lowering IOP if extensive irreversible changes in the trabecular meshwork have not already occurred. Trabeculectomy is usually a preferred choice for eyes with very high IOP and visual potential. However, in SO-associated glaucoma, trabeculectomy has low efficiency and high complication rates, and most authors advise searching for an alternative. For very high IOP, both glaucoma drainage devices and transscleral cyclophotocoagulation have proved to be safer and more efficient alternatives to trabeculectomy. Ex-PRESS mini shunt appears to be less efficient in terms of IOP lowering, but with an excellent safety profile. For less important IOP elevations, options such as supraciliary shunts, GATT and SLT may be useful, either alone or in conjunction with other methods.

## Figures and Tables

**Table 1:** Success rates and IOP evolution after AGV implantation.

**Study**	Success Rate at Timepoint	Preoperative IOP (Mean ± SD) ((mm Hg)	Postoperative IOP at Last Follow-Up (Mean ± SD) (mm Hg)	Definition of Success
Gupta et al. [41]	62% at 12 months	42.5 ± 6.63	18.3 ± 7.9	IOP ≤ 21 mm Hg without loss of light perception, additional glaucoma surgery or valve removal *
Al-Jazzaf et al. [47]	76% at 12 months 64% at 24 months	44 ± 11.8	14 ± 4.2	IOP between 5 and 21 mm Hg without loss of light perception or additional glaucoma surgery *
El-Saied et al. [44]	80% at 12 months	35 ± 4.7	11.2 ± 2.1	IOP ≤ 21 mm Hg and a reduction in IOP of ≥20% compared to preoperative IOP, with no additional glaucoma surgery or antiglaucoma topical medication
Ishida et al. [46]	70.2% at 24 months	39.1 ± 9.3	15 ± 7.9	IOP between 6 and 21 mm Hg without loss of light perception or additional glaucoma surgery *

* = with or without additional antiglaucoma medication or bleb needling; AGV = Ahmed glaucoma valve; IOP = intraocular pressure; SD = standard deviation.

**Table 2:** IOP and number of topical antiglaucoma medications evolution after TSCPC.

Study	TSCPC Type	Preoperative IOP (Mean ± SD) (mm Hg)	Postoperative IOP at Last Follow-Up (Mean ± SD) (mm Hg)	Preoperative Number of Antiglaucoma Medications (Mean or Mean ± SD)	Postoperative Number of Antiglaucoma Medications at Last Follow-Up (Mean or Mean ± SD)
Han et al. [22]	CW	43 ± 14.4	14.5 ± 4.3	2.6 ± 0.8	0.6 ± 1
Kumar et al. [54]	CW	34.5 ± 5.37	20.7 ± 4.49	3.38 ± 0.5	1.08 ± 0.8
Sivagnanavel et al. [53]	CW	39.6 ± 9.3	20 ± 13.5	2.6	1
Khodeiry et al. [52]	Slow coagulative CW	29.7 ± 9.6	14.6 ± 6.5	4.2 ± 0.9	1.9 ± 1.3

CW = continuous wave; IOP = intraocular pressure; SD = standard deviation; TSCPC = transscleral cyclophotocoagulation.

## Data Availability

Not applicable.
